# Performance during a strenuous swimming session is associated with high blood lactate: pyruvate ratio and hypoglycemia in fasted rats

**DOI:** 10.1590/1414-431X20187057

**Published:** 2018-03-26

**Authors:** P.B. Travassos, G. Godoy, H.M. De Souza, R. Curi, R.B. Bazotte

**Affiliations:** 1 Departamento de Farmacologia e Terapêutica, Universidade Estadual de Maringá, Maringá, PR, Brasil; 2 Departamento de Ciências Fisiológicas, Universidade Estadual de Londrina, Londrina, PR, Brasil; 3 Programa de Pós-Graduação Interdisciplinar em Ciências da Saúde, Universidade Cruzeiro do Sul, São Paulo, SP, Brasil

**Keywords:** Lactic acid, Lactate/pyruvate ratio, Exercise tolerance, Exhaustion, Gluconeogenesis

## Abstract

The aim of this study was to investigate the effect of lactatemia elevation and glycemia reduction on strenuous swimming performance in fasted rats. Three rats were placed in a swimming tank at the same time. The first rat was removed immediately (control group) and the remaining ones were submitted to a strenuous swimming session. After the second rat was exhausted (Exh group), the third one was immediately removed from the water (Exe group). According to the period of time required for exhaustion, the rats were divided into four groups: low performance (3–7 min), low-intermediary performance (8–12 min), high-intermediary performance (13–17 min), and high performance (18–22 min). All rats were removed from the swimming tanks and immediately killed by decapitation for blood collection or anesthetized for liver perfusion experiments. Blood glucose, lactate, and pyruvate concentrations, blood lactate/pyruvate ratio, and liver lactate uptake and its conversion to glucose were evaluated. Exhaustion in low and low-intermediary performance were better associated with higher lactate/pyruvate ratio. On the other hand, exhaustion in high-intermediary and high performance was better associated with hypoglycemia. Lactate uptake and glucose production from lactate in livers from the Exe and Exh groups were maintained. We concluded that there is a time sequence in the participation of lactate/pyruvate ratio and hypoglycemia in performance during an acute strenuous swimming section in fasted rats. The liver had an important participation in preventing hyperlactatemia and hypoglycemia during swimming through lactate uptake and its conversion to glucose.

## Introduction

Most studies evaluate metabolism alterations induced by fasting in resting conditions. However, mammals in nature face intense aerobic exercise in unexpected conditions and mechanisms for energy mobilization to ensure survival are activated even in the fasting state. On the other hand, studies on intense aerobic exercise in humans and in experimental animals are mostly performed in the fed state. However, there is a paucity of investigations about the metabolic changes induced by intense aerobic exercise associated with post-absorptive state.

After an overnight fast, fatty acids from adipose tissue and glucose from liver gluconeogenesis are the main energy sources. Aerobic exercise intensifies the catabolic condition of a fasting state in which there is increased intramyocellular triacylglycerol breakdown (1), fatty acids oxidation (2), and liver gluconeogenesis (3). Aerobic exercise performed in a fasted state increases further the rate of fat oxidation up to 24 h after the effort (4,5). Another characteristic of the short-term intense aerobic exercise in a fasted state is a reduction in glycemia (6) and elevation in lactatemia (7) associated with a shorter time to exhaustion. The reduction in performance is observed in fasted athletes during an acute physical exercise in comparison with fed ones (8,9). However, the high heterogeneity of these studies (such as exercise duration time, exercise intensity, sex of the participants, and training level of the participants), limit our capability to predict/discriminate the influence of blood glucose or lactate levels to promote exhaustion.

We previously reported that fasted rats, but not fed ones, submitted to an intensive forced swimming had the exhaustion time associated with hypoglycemia and hyperlactatemia (3). We also described that rats submitted to a strenuous exercise session have a great variability in time for exhaustion (3,10). A question was then raised: could this variability in time to exhaustion during a strenuous swimming exercise session be attributed to the reduction of glycemia, the elevation of blood lactate level or both? To evaluate these possibilities, fasted rats were divided into four groups according to the variability in time for exhaustion: low performance (3–7 min), low-intermediary performance (8–12 min), high-intermediary performance (13–17 min), and high performance (18–22 min). From the comparison of these four groups, the main purpose of this study was to investigate the correlation between the performance of the animals with blood levels of glucose, lactate, pyruvate, lactate/pyruvate ratio, and glycerol. Liver lactate uptake and its conversion to glucose during exercise and association with exhaustion were also evaluated.

## Material and Methods

### Animals

Male Wistar rats weighing 210–240 g were used in the experiments. The rats were maintained in a 22±1°C room with automatically controlled photoperiod (12 h light/12 h dark) and free access to water and commercial chow (Nuvilab¯, Brazil) until the day before the experiment when the animals were fasted for 15 h.

The swimming session was performed in cylindrical water tanks (60 cm height × 30 cm diameter) of 30 L capacity and water temperature at 31±1°C. The rats were kept in individual tanks. During swimming, the rats had a lead stone of 6% body weight tied to the tail. Exhaustion was defined as the incapacity to stay on the water surface, the loss of symmetrical movements during swimming or remaining underwater for more than 5 s (11).

The experimental protocol was approved by the ethical committee of the Universidade Estadual de Maringá and followed the international regulations on the protection of animals. For comparative purpose, we included not only a second rat without exercise (Control group), but also a third rat that was immediately removed from the water when the second rat reached exhaustion (Exh group). The exercised rat (Exe group) without exhaustion was included in order to differentiate the metabolic changes induced by the exercise (Exe group *vs* Control group) to the metabolic alterations caused by the exhaustion (Exe group *vs* Exh group).

### Experimental design

**Figure 1.**
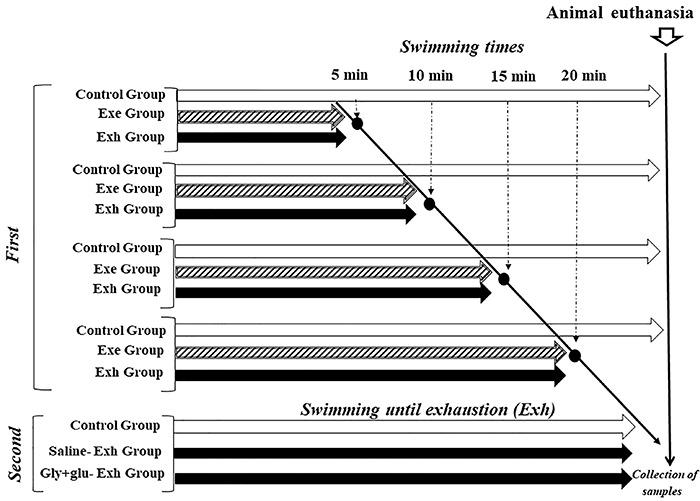
Design of the first and second experimental protocols. The control group was immediately removed from the tank. The exhausted (Exh) group was left until exhaustion, and the Exe group was immediately removed from the water at the same time as Exh. In the second experiment, the animals received oral (gavage) saline (1 mL) or 0.63 g/kg b.w. glycerol (Gly) + 0.25 g/kg b.w. glucose (glu), 20 min before starting the strenuous swimming session.

*First set of experiments* (Figure 1). Three rats were placed into the individual swimming tanks at the same time (total = 62 rats). The first rat was immediately removed (control group, n=5–8) and the remaining ones were left to swim. When the second rat was exhausted (Exh group), the third rat was also immediately removed from the water (Exe group). The rats that swam for less than 3 min or more than 22 min were excluded. The Exh group was subdivided into four subgroups: exhaustion between 3 and 7 min (Exh 5 min: low performance, n=5–8), exhaustion between 8 and 12 min (Exh 10 min: low-intermediary performance, n=4), exhaustion between 13 and 17 min (Exh 15 min subgroup: high-intermediary performance, n=4–8), or exhaustion between 18 and 22 min (Exh 20 min subgroup: high performance, n=4–8). The Exe group was subdivided in four subgroups: rats that swam between 3 and 7 min (Exe 5 min subgroup, n=5–7), 8 and 12 min (Exe 10 min subgroup, n=4), 13 and 17 min (Exe 15 min subgroup, n=4–8), or 18 and 22 min (Exe 20 min subgroup, n=4–7).

*Second set of experiments* (Figure 1). Three rats were placed into the individual swimming tanks at the same time (total = 12 rats). The first rat was removed immediately (control group, n=6) and the remaining two animals received oral (gavage) saline (1 mL) or glycerol (0.63 g/kg)+glucose (0.25 g/kg) 20 min before starting the swimming session. The rats that received saline (saline group, n=4–6) or glycerol plus glucose (n=5–6) were forced to swim until exhaustion.

### General procedure for all animals

All rats were removed from the swimming tanks and immediately killed by decapitation for blood collection or anesthetized for liver perfusion experiments.

### Blood samples and analysis

Blood was collected in tubes containing sodium EDTA and centrifuged at 1700 *g* for 10 min at 5°C. Plasma levels of glucose, pyruvate, lactate, and glycerol were measured as previously described (12).

### Liver perfusion experiments

Livers from the control (n=4–5), Exe (n=4–5) or Exh (n=4–5) groups were compared after hemoglobin-free liver perfusion. The rats were anesthetized with intraperitoneal ketamine (100 mg/kg b.w., União Química Farmacêutica Nacional S/A, Brazil) plus xylazine (10 mg/kg b.w., Bayer S/A, Brazil). After laparotomy, livers were perfused *in situ* without recirculation with Krebs-Henseleit buffer containing lactate (2 mM), pH 7.4, saturated with an O_2_/CO_2_ mixture (95/5%) pumped through a temperature regulated (37°C) membrane oxygenator prior to entering the liver via a cannula inserted into the portal vein (12,13). The whole procedure took about 10 min and the results obtained from these experiments reflect the *in vivo* condition immediately before the anesthesia, as previously described (3,10,12–14).

After 10 min of perfusion, lactate (2 mM) was dissolved in the perfusion fluid and infused between the 10–30 min of the perfusion period, followed by a period of post-infusion (10 min) to allow the return to basal levels. The activation of glucose and pyruvate production was measured as the difference between the rates of these metabolites released during (10–30 min) and before (0–10 min) the infusion of lactate. The differences allowed us to obtain and compare the areas under the curves (AUC). Liver lactate uptake was determined by the difference in the lactate concentrations during (10–30 min) and before (0–10 min) the infusion of lactate.

### Statistical analysis

Graph-Pad Prism program (GraphPad Software, USA) was used for statistical analysis. The data were assessed for normality using the Shapiro-Wilk test. The normally distributed data were analyzed using one-way ANOVA with the *post hoc* Tukey’s test for comparisons. The data that were not normally distributed were analyzed using the Kruskal-Wallis test with the Dunn test for *post hoc* comparisons. Differences between two means were analyzed using Mann Whitney-U for nonparametric tests. The Pearson product moment correlation was used to quantify the relationship among all variable estimated. Results are reported as means±SE. P<0.05 was used to indicate statistical significance.

## Results

### Blood levels of glucose, lactate, pyruvate, and lactate/pyruvate ratio

Blood levels of glucose, lactate, pyruvate, and lactate/pyruvate ratio after 5, 10, 15, or 20 min of forced swimming (Exe) or forced swimming until to exhaustion (Exh) are reported in Figure 1 (first set of experiments) and summarized in Table 1.

**Table 1.** Blood levels of glucose, lactate, pyruvate, and lactate/pyruvate ratio in 15 h fasted rats submitted to swimming and that reached exhaustion in 5, 10, 15, and 20 min.

	5 min	10 min	15 min	20 min
Glucose (mmol/L)				
Control	4.74±0.11 (n=8)	4.97±0.34 (n=4)	5.18±0.29 (n=8)	5.30±0.25 (n=7)
Exe	1.76±0.17 (n=8)^a^,^b^	2.28±0.14 (n=4)^a^	2.53±0.17^a^ (n=8)^a^	2.33±0.18 (n=7)^a^
Exh	3.14±0.16 (n=7)^a^	2.32±0.25 (n=4)^a^	1.28±0.08 (n=8)^a^,^b^	1.33±0.15 (n=8)^a^,^b^
Lactate (mmol/L)				
Control	2.67±0.75 (n=5)	2.27±0.13 (n=4)	2.31±0.21 (n=4)	2.61±0.49 (n=4)
Exe	7.69±0.79 (n=5)^a^	7.82±0.97 (n=4)^a^	4.65±0.27 (n=4)	6.44±0.70^a^ (n=4)^a^
Exh	13.3±0.42 (n=5)^a^,^b^	10.5±1.35 (n=4)^a^	9.54±1.26 (n=4)^a^,^b^	7.90±0.85^a^ (n=4)^a^
Pyruvate (mmol/L)				
Control	0.11±0.02 (n=5)	0.15±0.05 (n=4)	0.11±0.02 (n=4)	0.12±0.03 (n=4)
Exe	0.49±0.08 (n=5)^a^	0.51±0.08 (n=4)^a^	0.40±0.08 (n=4)^a^	0.34±0.08 (n=4)
Exh	0.20±0.04 (n=5)^b^	0.21±0.02 (n=4)^b^	0.23±0.06 (n=4)	0.25±0.05 (n=4)
Lactate/pyruvate ratio				
Control	25.1±4.7 (n=5)	23.5±3.1 (n=4)	21.9±2.9 (n=4)	25.4±5.9 (n=4)
Exe	19.7±2.6 (n=5)	19.4±5.8 (n=4)	13.4±6.6 (n=4)	23.3±6.9 (n=4)
Exh	74.9±4.9 (n=5)^a^,^b^	55.9±15.2 (n=4)	39.0±14.5 (n=4)	33.9±6.3 (n=4)

The control groups were placed into the water and removed immediately before starting swimming. Exhaustion (Exh) groups swam until exhaustion. Exercise (Exe) groups were removed from the water at the same time as the Exh animals Data are reported as means±SE.

a P<0.05 compared to the control group;

b P<0.05 compared to the Exe group (ANOVA).

Blood glucose levels were lower (P<0.05) after 5, 10, 15 or 20 min of the forced swimming (Exe or Exh groups *vs* Control group). In addition, except for 10 min of swimming, the reduction of glycemia was more pronounced (P<0.05) in the Exh group compared to the Exe group.

Except for 15 min of swimming, Exe group had higher (P<0.05) blood levels of lactate in comparison with the control group. The Exh group also had higher (P<0.05) blood concentrations of lactate in comparison with the Exe group for 5 and 15 min. In general, pyruvate levels were increased (P<0.05) during exercise (Exe *vs* Control group) and decreased (P<0.05) when forced swimming reached exhaustion (Exh *vs.* Exe group).

Lactate/pyruvate ratio remained unchanged during exercise (Exe *vs* Control group). However, lactate/pyruvate ratios were progressively lower from the group that reached exhaustion earlier (5 min) in relation to the group that reached exhaustion later (20 min).

### Correlations analysis

There was a correlation between the time to achieve exhaustion and the reduction of glycemia (P=0.0475) or elevation of lactatemia (P=0.0017). However, there was no correlation between forced swimming (Exe group) and the reduction of glycemia (P=0.1721) or elevation of lactatemia (P=0.0543) (Table 2).

**Table 2.** Correlation analysis between forced swimming (Exe group) or forced to swim up to exhaustion (Exh group) and glycemia or lactatemia.

Correlation	Confidence Interval (95%CI)	R^2^	P
Glycemia *vs* Time Exh	−0.79 to −0.00	−0.50	0.0475^a^
Lactatemia *vs* Time Exh	−0.89 to −0.34	−0.71	0.0017^a^
Glycemia *vs* Time Exe	−0.72 to 0.16	−0.35	0.1721
Lactatemia *vs* Time Exe	−0.79 to 0.00	−0.48	0.0543

a P<0.05.

### Liver lactate uptake and hepatic glucose and pyruvate production from lactate **-** first set of experiments

**Figure 2.**
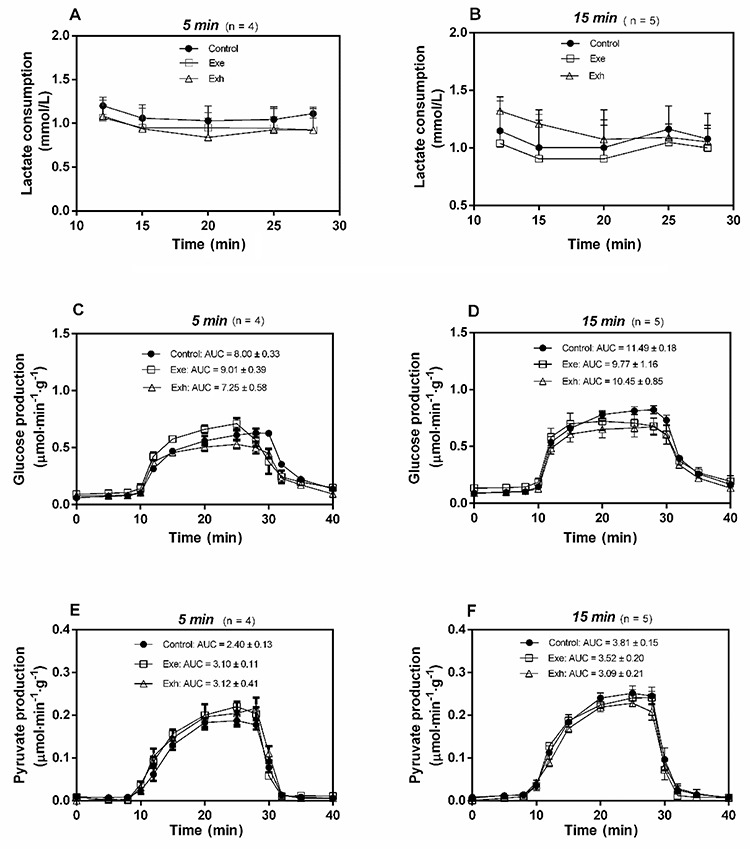
Lactate consumption (*A* and *B*), glucose (*C* and *D*), and pyruvate production (*E* and *F*) from lactate (2 mM) infused between 10 and 30 min, in the livers of 15-h fasted rats submitted to 5 or 15 min of forced swimming (Exe) or exhausted (Exh) after 5 or 15 min of forced swimming. The control group was placed in the water and removed immediately before starting swimming. Area under curves (AUC) is reported as µmol/g. Data are reported as means±SE.

The liver lactate uptake and the hepatic glucose and pyruvate production from lactate (2 mM) were similar in the Control, Exe, and Exh groups (Figure 2).

### Time of swimming and blood glucose, lactate, pyruvate, and glycerol levels – second set of experiments

Rats that received oral glycerol+glucose reached exhaustion later (P<0.05) in comparison with rats that received oral saline. The Saline Exh and Gly+glu-Exh groups had lower (P<0.05) glycemia in comparison with the sedentary control group while Gly+glu-Exh group had higher glycemia (P<0.05) in comparison with the Saline-Exh group. The Saline Exh and Gly+glu-Exh groups had higher (P<0.05) blood pyruvate and lactate levels in comparison with the control group whereas there was no difference for lactate and pyruvate levels compared to the Saline-Exh and Gly+glu-Exh groups. The Gly+glu-Exh groups had higher glycerol levels (P<0.05) in comparison with the control group or saline-Exh group (Table 3).

**Table 3.** Swimming time and blood glucose, lactate, pyruvate, and glycerol levels in 15-h fasted rats that received oral (gavage) saline (1 mL) or glycerol (0.63 g/kg)+glucose (0.25 g/kg), 20 min before the onset of forced swimming to exhaustion (Exh).

Parameters	Control	Saline Exh	P	Gly+glu Exh	P
Time Swimming (min)	–	12.6±1.7		19.0±1.0^b^	0.0238^a^
		(n = 6)		(n = 6)	
Glucose (mmol/L)	5.05±0.13	2.00±0.09^a^	0.0001^a^	2.54±0.09^a^,^b^	0.0001^a^
	(n = 6)	(n = 6)		(n = 6)	0.0103^b^
Lactate (mmol/L)	1.32±0.20	5.47±0.55^a^	0.0001^a^	6.3±0.41^a^	0.0001^a^
	(n = 6)	(n = 4)		(n = 5)	
Pyruvate (mmol/L)	0.07±0.01	0.18±0.01^a^	0.0002^a^	0.22±0.01^a^	0.0001^a^
	(n = 6)	(n = 5)		(n = 5)	
Glycerol (mmol/L)	0.12±0.013	0.19±0.011		98.2±22.49^a^,^b^	0.0002^a^,^b^
	(n = 6)	(n = 6)		(n = 6)	

The control groups were placed in water and removed immediately before starting swimming. Data are reported as means±SE.

a P<0.05 compared to the control group;

b P<0.05 compared to the saline group (ANOVA).

## Discussion

The capability of mammals to survive in conditions of prolonged fasting depends on the ability of the liver to produce energy by oxidizing fatty acids and use part of this energy to produce glucose. These metabolic processes are accelerated if fasting is associated with an intense aerobic physical exercise since there is an intensification of adipose tissue lipolysis and muscle glycolysis/proteolysis. As a consequence, blood concentrations of free fatty acids, glycerol, amino acids, and lactate are increased. With the increased supply of glucose precursors, particularly lactate, liver gluconeogenesis becomes a significant source of glucose during exercise.

In high-intensity aerobic exercise associated with fasting, a condition in which hepatic glycogen content is low, the glucose uptake and lactate production by skeletal muscles exceed lactate uptake and its conversion to glucose in the liver (15,16). In agreement with these studies, we found a correlation between hyperlactatemia and hypoglycemia with time to exhaustion in a strenuous swimming session. However, there is a time sequence in the participation of blood glucose and lactate for determining swimming performance. For rats that reached physical exhaustion between 3 and 7 min (Exh 5 min: low performance) or 8 and 12 min (Exh 10 min: low-intermediary performance), the time to exhaustion was better associated with higher lactate/pyruvate ratio. On the other hand, for rats that reached exhaustion between 13 and 17 min (Exh 15 min: high-intermediary performance) or 18 and 22 min (Exh 20 min: high performance), the exhaustion was better associated with hypoglycemia.

During an intense aerobic exercise session, lactatemia increases abruptly (17,18), resulting in elevation of the lactate/pyruvate ratio (indicative of the redox state - NADH:NAD^+^ ratio), as a consequence of the reduction of pyruvate to lactate through lactate dehydrogenase (19). In this condition, the brain could receive a considerable lactate supply. However, there is a limitation in the transport of lactate through the blood-brain barrier and the elevated blood lactate levels cannot supply the brain lactate deficit due to hypoglycemia (20). In fact, the energy homeostasis in the brain depends on the metabolic cooperation between astrocytes and neurons. Thereby, the energy metabolism in astrocytes is predominantly glycolytic and lactate produced from glucose is released and then used by the neurons (21). Thus, the hypothesis that during acute exercise-induced hypoglycemia there is a short-term deficit of glucose availability in astrocytes and consequently a lactate deficit in neurons that cannot be compensated by the elevation of blood lactate levels is confirmed.

Stressful conditions, with increased sympathetic activity, are commonly associated with hyperglycemia (22). However, we reported that strenuous swimming is associated with hypoglycemia in fasted rats. Hypoglycemia associated with intensive exercise is also reported in diabetic patients receiving insulin (23).

To confirm that energy supply influences performance during physical exercise in fasted rats, glycerol plus glucose was used as energy precursors. Animals that received oral glycerol plus glucose before swimming had better performance, i.e., the exhaustion occurred later in comparison with animals that received saline before exercise. The contribution of glycerol to performance may be attributed not only to its hepatic conversion to glucose during exercise (3), but also by the fact that glycerol is directly used by skeletal muscle and its uptake increases by many folds during exercise (24). It must be emphasized that blood lactate levels and liver glucose production from lactate play a central role for glycemia and lactatemia maintenance during exercise (3,25). Lactate is converted into glucose through various steps in the liver gluconeogenesis pathway.

During liver perfusion experiments, infused lactate crosses the cell membrane and is then converted to pyruvate in the cytosol. Pyruvate then enters into the mitochondria where it is carboxylated and then leaves the mitochondria as malate. In cytosol, malate is converted to oxaloacetate and then to phosphoenolpyruvate and, after various steps, is converted by microsomal glucose-6-phosphatase to glucose, which is released from the liver to the blood.

Liver lactate uptake and glucose production from lactate remained unchanged during strenuous exercise (Exercise group and Exhausted group). The liver prevents plasma lactate elevation and blood glucose decrease during an acute intense swimming section through lactate uptake and its conversion to glucose, corroborating studies by others (26,27).

A limitation of the experiments in isolated perfused liver is the use of a unique concentration of lactate, i.e. 2 mM, whereas *in vivo* plasma lactate concentration exhibits great variability in the sedentary condition and in the strenuous exercise associated with exhaustion (28).

Glucose from liver gluconeogenesis is used for ATP production in skeletal muscle through glycolysis, generating lactate and pyruvate (29). During skeletal muscle contraction, the activation of the AMP-activated protein kinase (AMPK) in response to the increased ATP demand (30) favors the translocation of type 4 glucose transporter (GLUT 4) to the plasma membrane increasing glucose uptake (31–34), which is associated with decreased glycemia and the consequent hypoglycemia (35).

In conclusion, there was a time sequence in the participation of lactate/pyruvate ratio and hypoglycemia determining the performance during an intense aerobic swimming section in fasted rats. Furthermore, the liver had an important participation in the prevention of hyperlactatemia and hypoglycemia during swimming through lactate uptake and its conversion to glucose.
